# Changes in Bull Semen Metabolome in Relation to Cryopreservation and Fertility

**DOI:** 10.3390/ani10061065

**Published:** 2020-06-19

**Authors:** Valentina Longobardi, Michal A. Kosior, Nunzia Pagano, Gerardo Fatone, Alessia Staropoli, Anastasia Vassetti, Francesco Vinale, Giuseppe Campanile, Bianca Gasparrini

**Affiliations:** 1 Department of Precision Medicine, University of Campania Luigi Vanvitelli, 80138 Naples, Italy; longobardivalentina@gmail.com; 2 Department of Veterinary Medicine and Animal Production, Federico II University of Naples, 80137 Naples, Italy; m.kosior@hotmail.com (M.A.K.); nunzia91n@libero.it (N.P.); francesco.vinale@ipsp.cnr.it (F.V.); giucampa@unina.it (G.C.); bgasparr@unina.it (B.G.); 3 CNR Institute for Sustainable Plant Protection, 80055 Portici, Italy; al.staropoli@gmail.com (A.S.); an.vassetti@libero.it (A.V.)

**Keywords:** fertility, bull, LC–MS, cryopreservation, metabolites

## Abstract

**Simple Summary:**

Although semen cryopreservation has facilitated the diffusion of artificial insemination and in vitro fertilization in cattle, it still represents a major factor affecting sperm fertility. It is known that cryopreservation induces the loss of fertility-associated proteins, while the effect on metabolites has not been evaluated, although several compounds affect sperm physiology and fertility. The aim of the present work was to study the metabolome in bovine sperm and seminal plasma after cryopreservation and to correlate the metabolic profile of high- and low-fertility bulls in order to identify fertility markers. The analysis, carried out by liquid chromatography–mass spectrometry methods, revealed differences in metabolite contents between fresh and cryopreserved semen, both at cellular and plasmatic levels. Interestingly, metabolites showing variation have important functions related to fertility. In addition, the study highlighted the differences in lipid profile between high- and low-fertility bulls. The identification of new potential fertility markers is of high economic impact. In addition, it opens the way for the development of corrective strategies to improve the fertility of low-fertility bulls.

**Abstract:**

Semen cryopreservation determines several sperm damages, including the loss of fertility-associated proteins. The purpose of the study was to compare the metabolite contents in bovine sperm and seminal plasma before and after cryopreservation, and between high- and low-fertility bulls in vitro. Forty-eight ejaculates, collected from eight bulls (six per bull), were analyzed by liquid chromatography–mass spectrometry. Cryopreservation resulted in an over-expression of lysophosphatidylcholine (0:0/18:2(9Z,12Z)) in seminal plasma. In addition, higher levels of glycine betaine and pyro-l-glutaminyl-l-glutamine were observed in cryopreserved compared to fresh spermatozoa. The fresh seminal plasma of high-fertility bulls showed an over-expression of l-acetylcarnitine, glycerol tripropanoate, 2,3-diacetoxypropyl stearate and glycerophosphocholine, and an under-expression of lysophosphatidylcholine and butyrylcarnitine, compared to low-fertility bulls. Higher levels of glycerophosphocholine and lysophosphatidylcholine (16:0/0:0) were recorded in fresh spermatozoa from high-fertility bulls. In high-fertility bulls, a greater content of glycerophosphocholine and lower levels of butyrylcarnitine, glycine betaine and l-carnitine were found in cryopreserved seminal plasma, and lower levels of glycine betaine were detected in cryopreserved spermatozoa. In conclusion, cryopreservation affects bovine semen metabolome at both plasmatic and cellular compartments, and metabolic profile differs between high- and low-fertility bulls.

## 1. Introduction

Fertility is a major factor influencing the sustainability of livestock breeding. It is known that reproductive failure in cattle is in great part due to the poor fertilizing ability of sperm [1,2]. Semen cryopreservation has played a fundamental role in spreading the use of advanced reproductive technologies, such as artificial insemination (AI) and in vitro fertilization (IVF) in cattle. Nevertheless, cryopreservation is an important factor affecting sperm fertility. Indeed, freezing–thawing can induce several sperm damages [3], the premature capacitation of spermatozoa [4,5] and the loss of fertility-associated molecules [6,7]. Indeed, it is known that freezing–thawing induces the leakage of proteins, especially enzymes, from inside the spermatozoa to the extracellular fluid [8,9]. In addition, it was demonstrated that the proteomic profile of bovine seminal plasma is affected by cryopreservation [10].

Another important limiting factor is the bull effect (i.e., the high individual variability in fertilizing ability recorded among bulls) [6,11]. Despite an adequate production of motile and morphologically normal sperm, some bulls have low fertility, whose molecular mechanisms are not known [12]. Therefore, the ability to predict bull fertility in advance offers enormous benefits for the economic success of livestock enterprise by improving pregnancy rates [13]. Currently, the assessment of bull fertility still relies on several fertility tests, including motility, viability and morphology; however, while these conventional analyses allow for the detection of unsuitable ejaculates, they often fail to accurately predict real fertility.

Metabolomics characterizes the downstream of biology systems and has been recently applied to study reproductive processes [14,15,16]. This approach allows for the comprehensive identification of metabolites, such as amino acids, peptides, fatty acids and carbohydrates in secretions, cells, tissues and organs [17,18,19]. Metabolomics has been recently used to identify male fertility biomarkers in farm animals, including cattle [20,21]. Seminal plasma has long been used as a key source to investigate male infertility. The metabolites of seminal plasma can affect many aspects of sperm physiology and influence bull fertility, by changing gene and protein expression [22]. Metabolomic profiling has been used to identify potential fertility biomarkers in the seminal plasma of bulls [15] and men [14]. In cattle, the analysis of seminal plasma by gas chromatography–mass spectrometry (GC–MS) allowed for the identification of sixty-three metabolites [21]. In the same study, a higher concentration of 2-oxoglutaric acid and fructose were found in bulls classified as high-fertile, suggesting the potential use of metabolites as biomarkers of bull fertility [21]. A variety of techniques, such as mass spectrometry, nuclear magnetic resonance spectroscopy, and Fourier transform infrared spectroscopy, are available in metabolomics at present [19]. Among them, liquid chromatography–mass spectrometry (LC–MS) has already been used in metabolomics studies of seminal plasma in humans [23].

To our knowledge, the variation in metabolite contents following semen cryopreservation has still not been investigated in cattle. Metabolites are reliable indicators of phenotypic traits, as they are the end products of metabolic pathways [15]. The identification of fertility-associated metabolites can open the way for the development of corrective strategies, by reintegrating key compounds lost during cryopreservation to restore the fertility of cryopreserved semen.

The aim of this study was to evaluate the differences in the metabolites present in seminal plasma and spermatozoa between fresh and cryopreserved bovine sperm by a metabolomic approach (LC–MS). A further objective of this work was to compare the metabolomes of seminal plasma and spermatozoa of high-fertility (HF) and low-fertility (LF) bulls, as assessed in vitro, to identify new reliable fertility markers.

## 2. Materials and Methods

### 2.1. Experimental Design

In this work both the seminal plasma and spermatozoa of fresh and cryopreserved semen were analyzed. Eight healthy Holstein (Bos Taurus) bulls (4–6 years of age), maintained at an authorized National Semen Collection Center (Centro Tori Chiacchierini, Civitella D’Arna, Perugia, Italy; authorization numbers: PG0001C for Italy and IT014 for Europe), under uniform management conditions and routinely used for semen collection, were selected for the trial. For this study, a total of forty-eight ejaculates were collected weekly from eight bulls (six ejaculates per bull) by artificial vagina (IMV, L’Aigle, France). On fresh semen, motility was evaluated by phase contrast microscopy and only ejaculates containing more than 80% motile sperm were used in the study. After the initial semen assessment (volume, concentration and motility), each ejaculate was split into two aliquots. An aliquot was centrifuged (3000 rpm for 10 min, at 25 °C) to separate seminal plasma and spermatozoa and were immediately processed for analysis (fresh seminal plasma and spermatozoa, termed F-P and F-S groups, respectively). The other aliquot was cryopreserved by standard method with commercial extender BioXcell (IMV-technologies, L’Aigle France), to have a final concentration of 30 × 10^6^ spermatozoa per mL in 0.5 mL French straws, which underwent combined cooling with an equilibration period of 3 h at 5 °C. The straws were kept in an automatic programmable biological cell freezer (IMV technology, France) until the temperature of straws reached −145 °C. Then, the straws were plunged into liquid nitrogen (−196 °C) for storage. The straws were thawed at 37 °C for 40 s in a water bath, and the seminal plasma and spermatozoa were separated by centrifugation (3000 rpm for 10 min, at 25 °C) and stored at −20 °C (for approximately two months) until further analysis (cryopreserved seminal plasma and spermatozoa, termed CRY-P and CRY-S groups, respectively). In order to distinguish HF and LF bulls, sperm viability, morphology, membrane integrity, DNA fragmentation, as well as in vitro fertilizing ability, were evaluated after thawing.

### 2.2. Assessment of Fertility Parameters

Cryopreserved semen straws from the same ejaculates (*n* = 48) used for metabolite extraction were thawed and utilized for the assessment of sperm viability, morphology, membrane integrity and DNA fragmentation. Cleavage and blastocyst rates were evaluated after in vitro fertilization with frozen–thawed spermatozoa from three randomly selected ejaculates/bull (*n* = 24).

Sperm viability (percentage of acrosome intact live sperm) and morphology were assessed by the Trypan Blue/Giemsa technique, as previously described [5]. The functional integrity of the sperm was assessed by the Hypo osmotic swelling (HOS) test [5] and expressed as the percentage of sperm showing swollen tails (HOS+ sperm) following incubation in a hypoosmolar solution of 150 mOsm/L. DNA fragmentation index was assessed by Tunel assay [24] using a commercially available kit (In Situ Cell Death Detection Kit, Roche, Indianapolis, IN, USA). The sperm were analyzed by using a fluorescent microscope (Eclipse E−600; Nikon, Japan) under ultraviolet light with excitation DAPI (460 nm for blue fluorescence), and FITC (520 nm for green fluorescence) filters and the numbers of total (blue) and Tunel–positive (green) nuclei were recorded. At least 200 spermatozoa were analyzed for viability, membrane integrity and DNA fragmentation assessments.

To assess in vitro fertilizing ability, abattoir-derived bovine oocytes were matured and fertilized in vitro with frozen–thawed sperm from different bulls, then cultured in vitro to the blastocyst stage, according to our standard procedure [25]. Briefly, cumulus–oocyte complexes (COCs) were aspirated from follicles of 2–8 mm in diameter and only those with uniform cytoplasm and multilayered cumulus cells were matured in TCM 199, supplemented with 15% bovine serum (BS), 0.5 µg/mL FSH, 5 µg/mL LH, 0.8 mM l-glutamine and 50 µg/mL gentamycin (25–30 COCs/400 µL) in four well plates (NuncTM, Roskilde, Denmark) for 22 h at 39 °C and 5% CO_2_ in the air. In vitro, matured COCs were washed and transferred into 300 µL of Tyrode’s modified medium without glucose and bovine serum albumin (BSA), supplemented with 5.3 SI/mL heparin, 30 µM penicillamine, 15 µM hypotaurine, 1 µM epinephrine and 1% BS (IVF medium). The sperm selected by BoviPure gradients (according to the manufacturer’s instructions, Nidacon, Sweden) were diluted with IVF medium and added in the fertilization wells at a concentration of 1 × 10^6^ sperm/mL. Gametes were co-incubated for 20 h at 39 °C in 5% CO_2_ in the air, after which presumptive zygotes were vortexed for 2 min to remove cumulus cells in Hepes–TCM with 5% BS, washed twice in the same medium and randomly distributed into 400 µL Synthetic Oviduct Fluid (SOF) medium with 30 µL/mL essential amino acids, 10 µL/mL nonessential amino acids and 5% BS and incubated in a humidified mixture of 5% CO_2_, 5% O_2_ and 88% N_2_ in the air at a temperature of 39 °C. The cleavage and blastocyst rates, calculated out of the total COCs, were recorded, respectively, on days two and seven after in vitro fertilization.

### 2.3. Metabolite Extraction

Metabolites extraction was performed by adding 900 µL methanol to 300 µL seminal plasma, vortexed for 1 min and stored at −20 °C for 2 h, in order to precipitate the proteins. The samples were then centrifuged at 12,000 rpm for 15 min at 4 °C and the supernatant was filtered with 0.22 µm membrane syringe filters. Spermatozoa were lysed in methanol according to a protocol previously described [26], consisting briefly in eight long-lasting pulses of 100% amplitude and 10 kHz in a sonicator Vibra-Cell™ (Sonics & Materials, Inc., Newtown, CT, USA). To avoid specimen heating, the samples were kept on ice while sonicating. Following this step, the samples were kept on ice for a further 30 min and then the same protocol used for seminal plasma was followed. Unless otherwise stated, the reagents were purchased from Merck/Sigma-Aldrich (Milano, Italy).

### 2.4. LC–MS Analysis

Analyses were done on an Agilent high performance liquid chromatograph (HPLC) 1260 Infinity Series (Agilent Technologies, Santa Clara, CA, USA) equipped with a DAD (Diode Array Detector) system (Agilent Technologies) and coupled to a quadrupole-time of flight (Q-TOF) mass spectrometer model G6540B (Agilent Technologies) with a Dual ESI source (Agilent Technologies). The column used for separation was an Ascentis^®^ Express C-18 column (2.7 μm, 50 mm × 3.0 mm i.d., Supelco©, Bellefonte, PA, USA), held at a constant temperature of 40 °C. The mobile phase consisted of A: 0.1% (*v*/*v*) formic acid (FA) in water (H_2_O) and B: 0.1% formic acid (FA) in acetonitrile (ACN). Elution was done at a flow rate of 0.6 mL/min and the gradient was as follows: starting condition 5% B, held for 1.10 min, ramping to 95% B until 9.30 min, held at 95% B for 1.10 min, lowering to 5% B in 2.30 min, and 5% B for 3 min as equilibration time. The injection volume was 4 µL. UV spectra were collected by DAD, setting the detection wavelength at 210, 250 and 280 nm. Both HPLC and MS and their parameters were set using the Agilent MassHunter Data Acquisition Software, rev. B.05.01 (Agilent Technologies). The system operated in positive ion mode and MS spectra were recorded in the *m*/*z* 50–1000 range as centroid spectra, with a speed of 3.3 spectra/s. The capillary was maintained at 2000 V, fragmentor voltage at 180 V, cone 1 (skimmer 1) at 45 V and Oct RFV at 750 V. The gas flow rate was set at 11 L/min at 350 °C and the nebulizer was set at 45 psig. A standard solution was infused by using an Isocratic pump (1260 Infinity Series, Agilent Technologies) in order to perform the real-time lock mass correction. The solution consisted of two reference mass compounds: purine (C_5_H_4_N_4_ at *m*/*z* 121.050873, 10 µmol/L) and hexakis (1H, 1H, 3H-tetrafluoropentoxy)–phosphazene (C_18_H_18_O_6_N_3_P_3_F_24_ at *m*/*z* 922.09798, 2 µmol/L). The flow rate was set at 0.06 mL/min, while the detection window and the minimum height were set at 1000 ppm and 10.000 counts, respectively, for reference mass correction.

#### LC–MS Raw Data Analysis

Data from the LC–MS analysis were first subjected to the extraction of peak components and to chromatographic alignment, performed by using MassHunter Profinder, version 06.00. The batch recursive feature extraction was used to extract and align the molecular features in all biological and analytical replicates for each group of samples. The parameters for molecular feature extraction included a peak height of ≥ 5000 counts and the extraction of possible ions inclusive of the protonated ion, or the sodiated adduct. At least two ions were required for a single molecular feature. For binning and alignment, a tolerance of 0.3 min and 20 ppm was set for the retention time window and mass window, respectively. The post-processing filters applied included the requirement for the molecular feature to be present in at least three out of six replicates in one group.

### 2.5. Statistical Analysis

Statistical analysis was carried out using Mass Profile Professional, version 13.1.1 (Agilent Technologies). Aligned data on seminal plasma and spermatozoa from fresh (F-P and F-S, respectively), and cryopreserved (CRY-P and CRY-S, respectively) groups were filtered based on the quality of their presence (present–marginal–absent) in the samples and conditions. After this operation, only entities in which at least 100% of the values in any one of two conditions have acceptable values, were retained. The remaining entities were further filtered based on their frequency of occurrence among the samples and conditions. In particular, only entities that appeared in at least 100% of the samples, in at least one condition, were retained. An unpaired Student *t*-test (*p* > 0.05) with Tukey–HSD post hoc and Benjamini–Hochberg correction was applied to assess the differential significance of samples. Eventually, fold change > 2.0 was applied. The results obtained were then subjected to principal components analysis (PCA) and hierarchical clustering in order to compare the metabolome of seminal plasma and spermatozoa between HF and LF bulls to detect differences in metabolome induced by the cryopreservation.

Statistically relevant compounds were identified using the freely available electronic database, Human Metabolome database (HMDB) and by comparison with standard compounds or with data present in the literature. Among the detected molecules, only those with a mass error below 10 ppm and a sufficient score (≥95%) were reported.

## 3. Results

### 3.1. Metabolomic Analysis of Seminal Plasma before and after Cryopreservation

**Figure 1:**
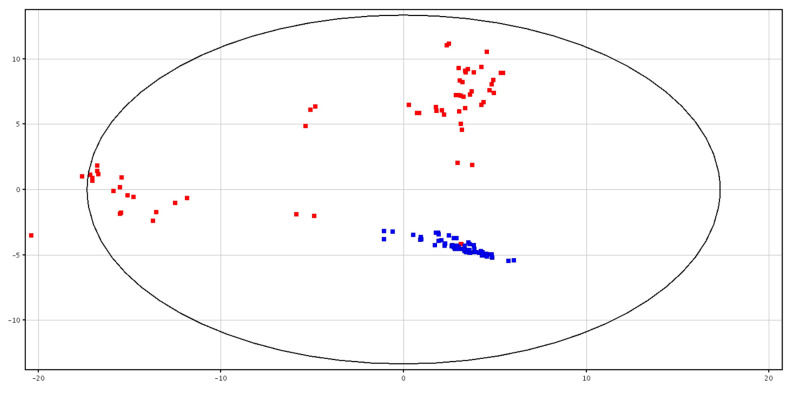
PCA scores plots of the LC–MS data acquired for fresh (in blue; F-P) and cryopreserved (in red; CRY-P) seminal plasma. PC1 (*x*-axis) is 37.3%; PC2 (*y*-axis) is 22.22%.

The metabolic profile was different between fresh (F-P) and cryopreserved (CRY-P) seminal plasma. The separation is shown in PCA scores plot, as shown in Figure 1. Hierarchical clustering analysis also showed a separation of these groups, related to a different chemical composition, as shown in Figure S1. In particular, a putatively identified metabolite was under-expressed in the F-P samples, as shown in Table 1.

### 3.2. Metabolomic Analysis of Spermatozoa before and after Cryopreservation

**Figure 2:**
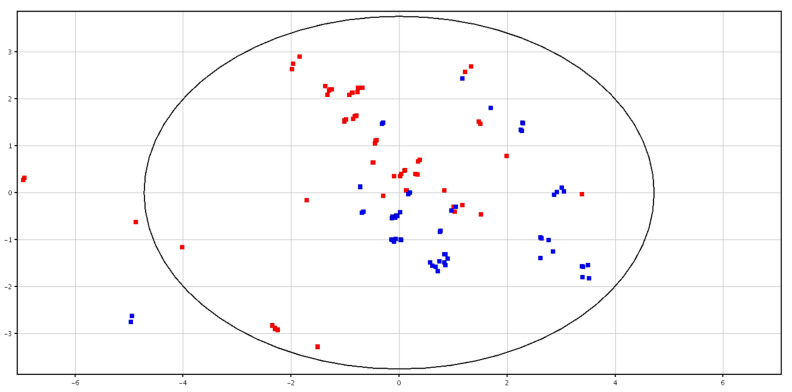
PCA scores plots of the LC–MS data acquired for fresh (in blue; F-S) and cryopreserved (in red; CRY-S) spermatozoa. PC1 (*x*-axis) is 32.23%; PC2 (*y*-axis) is 20.35%.

Differences between fresh and cryopreserved spermatozoa were detected, as shown in Figure 2. Clustering for the two groups showed the total variance within the PCA data, as shown in Figure S2. In particular, two putatively identified metabolites (i.e., glycine betaine and pyro-l-glutaminyl-l-glutamine), were higher in content in the cryopreserved samples, as shown in Table 1.

### 3.3. Metabolomic Analysis of Seminal Plasma from HF and LF Bulls

The fertility-associated parameters of HF and LF bulls are shown in Table 2.

**Figure 3:**
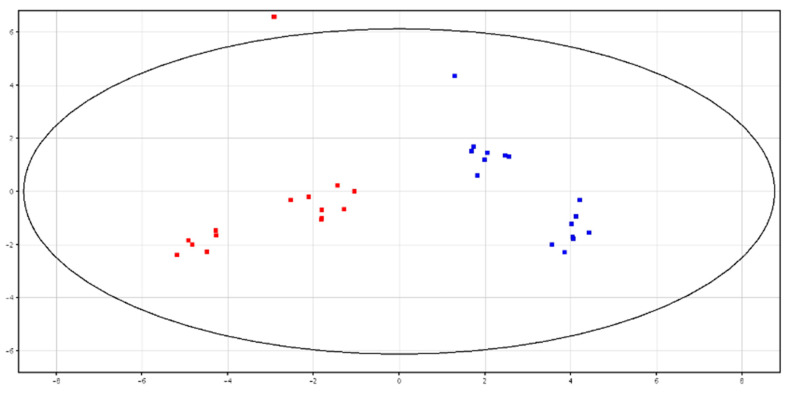
PCA scores plots of the LC–MS data acquired from fresh seminal plasma for high (in red; HF) and low (in blue; LF) fertility bulls. PC1 (*x*-axis) is 43.77%; PC2 (*y*-axis) is 21.53%.

**Figure 4:**
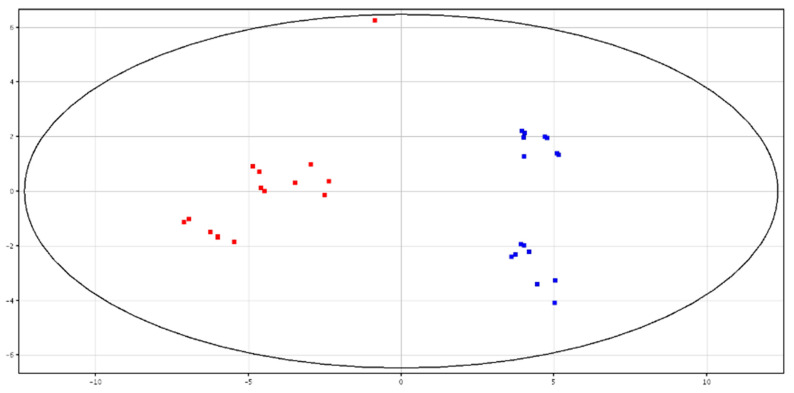
PCA scores plots of the LC–MS data acquired from cryopreserved seminal plasma for high (in red) and low (in blue) fertility bulls. PC1 (*x*-axis) is 50.89%; PC2 (*y*-axis) is 13.96%.

Principal Component Analysis (PCA)and hierarchical clustering showed a distinct separation between HF and LF bulls, according to the metabolic content in fresh and cryopreserved seminal plasma, as shown in Figure 3 and Figure 4 and Figures S3 and S4. All of the identified metabolites are reported in Table 3. High-fertility bulls showed higher levels of l-acetylcarnitine, glycerol tripropanoate, 2,3-diacetoxypropyl stearate and glycerophosphocholine (GPC), and lower levels of butyrylcarnitine, lysoPC (P-16:0/0:0) and piperidine in the F-P samples. In the CRY-P samples, higher levels of GPC and lower levels of l-carnitine, butyrylcarnitine and glycine betaine were found in HF bulls.

### 3.4. Metabolomic Analysis of Spermatozoa from HF and LF Bulls

**Figure 5:**
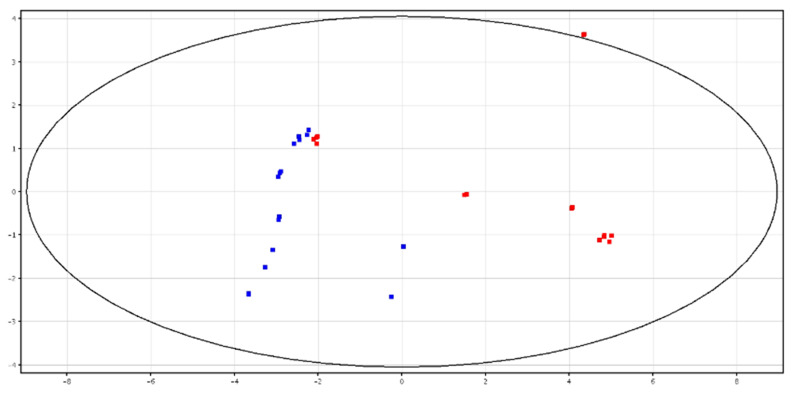
PCA scores plots of the LC–MS data acquired from fresh spermatozoa for high (in red) and low (in blue) fertility bulls. PC1 (*x*-axis) is 54.94%; PC2 (*y*-axis) is 13.29%.

**Figure 6:**
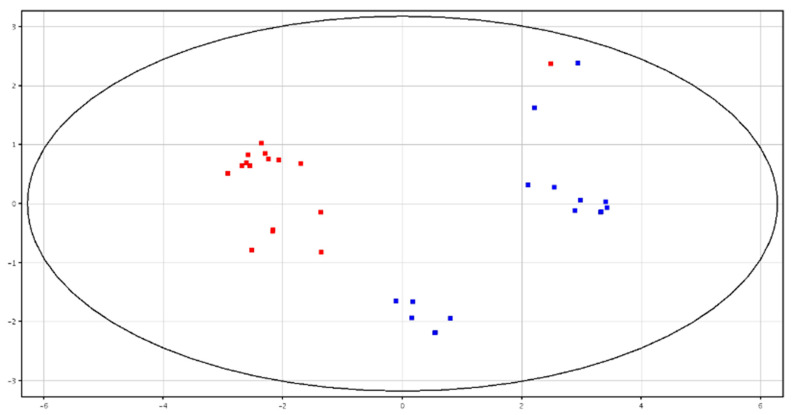
PCA scores plots of the LC–MS data acquired from cryopreserved spermatozoa for high (in red; HF) and low (in blue; LF) fertility bulls. PC1 (*x*-axis) is 59.92%; PC2 (*y*-axis) is 15.38%.

Both PCA and hierarchical clustering differentiated HF and LF bulls according to the metabolite contents in fresh and cryopreserved spermatozoa, as shown in Figure 5 and Figure 6 and Figures S5 and S6. All of the identified compounds are reported in Table 3.

## 4. Discussion

This study analyzed, using LC–MS techniques, the changes in metabolite contents in bovine sperm and seminal plasma occurring during cryopreservation. A further objective was to compare the metabolite contents in fresh and cryopreserved sperm and seminal plasma between HF and LF bulls, in order to identify fertility markers. To our knowledge, this is the first report showing a variation in the metabolome of bull seminal plasma and spermatozoa due to cryopreservation.

### 4.1. Metabolomic Analysis of Seminal Plasma before and after Cryopreservation

The results of the study showed that cryopreservation influences the metabolome of seminal plasma. Indeed, in the diluted seminal plasma obtained from semen cryopreserved by conventional freezing (CRY-P), some compounds were in higher amount, compared to fresh seminal plasma (F-P) among which LysoPC (0:0/18:2(9Z,12Z)) was putatively identified. LysoPC (0:0/18:2(9Z,12Z)) is a lysophospholipid characterized by the presence of one chain of linoleic acid at the C-2 position. It is formed by the hydrolysis of phosphatidylcholine by the enzyme phospholipase A2 as part of the de-acylation/re-acylation cycle. Lysophosphatidylcholine has proinflammatory properties, cell signalling functions and is known to induce sperm capacitation and acrosome reaction by increasing Ca^2+^ concentrations [27,28]. Therefore, the higher content of lysoPC detected in cryopreserved seminal plasma may at least in part account for the capacitation-like changes (cryo-capacitation) commonly observed following cryopreservation, due to increased oxidative stress [5]. In this regard, lysophosphatidylcholine has been proven to be a reliable marker of the lipid oxidation of sperm membrane [29]. This is important because cryo-capacitation results in a decrease in the reproductive lifespan of the spermatozoa [30] that may impair fertility, particularly after AI. Furthermore, a premature acrosome reaction undoubtedly causes a loss of fertility [4]. However, whether the lysophosphatidylcholine increase in CRY-P is due to the cryopreservation process or to the extender composition is not known. Therefore, the confounding effect of the extender may impede the drawing of definitive conclusions, making it difficult to hypothesize potential corrective strategies.

### 4.2. Metabolomic Analysis of Spermatozoa before and after Cryopreservation

The metabolome of spermatozoa derived from cryopreserved semen (CRY-S) did not show many differences compared to fresh spermatozoa. Indeed, only two metabolites (i.e., glycine betaine and pyro-l-glutaminyl-l-glutamine) were higher in CRY-S. This suggests that conventional semen cryopreservation is efficient to protect sperm from cryoinjuries to a certain extent. Among the two putatively identified metabolites, glycine betaine is of particular interest. Glycine betaine is a methyl group donor that functions in the normal metabolic cycle of methionine—transforming the harmful homocysteine into methionine [31]—modulates cellular responses to osmotic stress [32], acts as an osmolyte and cryoprotectant [33] and exerts antioxidant and anti-inflammatory actions [34,35]. It has been reported that betaine improves the quality of cryopreserved spermatozoa in ram [36,37] and stallions [38,39]. The supplementation of betaine to the extender improved semen quality in boar [40]. In cattle, the replacement of glycerol with betaine improved motility when spermatozoa were stored at 20 °C, 5 °C and 0 °C [41]. The higher amount of glycine betaine in cryopreserved spermatozoa in this study is difficult to interpret, given the limits of nontargeted metabolomics, but different hypotheses can be put forward. It is not possible to know whether this is due to reduced utilization or to increased production from choline oxidation. In favor of the first hypothesis, there is the known slowing-down of sperm metabolism induced by cryopreservation [3]. However, it is also recognized that, during dilution, cooling–freezing and thawing procedures, sperm are faced with physiological and structural challenges due to changes in osmotic balance, oxidative stress and the formation of intracellular ice crystals [3]. Therefore, we speculate that the higher levels of glycine betaine in cryopreserved spermatozoa may be more likely due to a cellular adaptative response to cryopreservation-induced oxidative and osmotic stress. Indeed, in addition to the antioxidant function [34,35], betaine is a compatible osmolyte, known to accumulate in the cell cytoplasm to prevent osmotic stress [42]. It follows that the higher amounts of the metabolite may result from the attempt of spermatozoa to counteract cryopreservation-induced stress [3]. The finding is undoubtedly interesting and hence it will be worth carrying out in vitro studies in the future to validate the hypotheses made.

### 4.3. Metabolomic Analysis of Seminal Plasma from HF and LF Bulls

The fresh seminal plasma of HF bulls showed a different lipid profile compared to LF bulls, with an accumulation of l-acetylcarnitine, glycerol tripropanoate, 2,3-diacetoxypropyl stearate and GPC, and a depletion of lysoPC (P-16:0) and butyrylcarnitine.

Particularly interesting is the accumulation in fresh seminal plasma of HF bulls of l-acetylcarnitine, the most abundant and powerful of the acylcarnitine derivates. l-carnitine is an hydrosoluble amino acid that plays a critical role as a cofactor essential for fatty acid metabolism, by transporting fatty acids into the mitochondria for subsequent β-oxidation and hence energy generation. This process results in the esterification of l-carnitine to produce acylcarnitines, among which the l-acetylcarnitine is the most predominant. In addition, l-carnitine plays a critical role in modulating the intracellular CoA homeostasis. Carnitine is present in high concentration in the epididymis and in the spermatozoa of several species [43,44,45,46]. During transit in the epididymis, bovine sperm accumulate carnitine that is rapidly acetylated [47]. Acetylcarnitine/l-carnitine may act as a carrier system of acetyl groups between the mitochondria and cytoplasm via a translocase present on the mitochondrial membrane, playing an important role for sperm metabolism [48]. It was also reported that l-acetylcarnitine levels and a high l-acetylcarnitine:free l-carnitine ratio in human spermatozoa were associated with motility [49,50]. Semen carnitine levels are also correlated with sperm concentration and progressive motility in stallions [51]. Finally, the supplementation of the semen extender with carnitine was demonstrated to improve sperm quality in cattle and buffalo [5,52]. The importance of this compound and its role on fertility are related to its antioxidant and anti-inflammatory functions [53,54], as well as to the regulative action on sperm metabolism [48]. It follows that the higher amount of l-acetylcarnitine in the fresh seminal plasma of HF bulls suggests that it can be considered a male fertility biomarker.

In addition, the triacylglycerols, glycerol tripropanoate and 2,3-diacetoxypropyl stearate, were accumulated in HF bulls. It was previously demonstrated that sperm can utilize triglycerides for their metabolism [55,56]. It is known that seminal plasma contains cholesterol and triacylglycerols, the latter serving as energy sources [57]. A variation in the lipid composition of seminal plasma in relation to season and semen quality was observed in cattle, with higher cholesterol levels related to better semen quality [58]. The fatty acid content of seminal plasma was correlated to semen quality in bulls and stallions [58,59]. Higher levels of cholesterol and triacylglycerols were associated to fertility parameters in cats [60]. In contrast, triacylglycerol levels were greater in the seminal plasma of azoospermic men [61]. Furthermore, the higher levels of GPC in the seminal plasma of HF bulls are in agreement with a previous study, revealing that seminal plasma GPC is a marker of fertility in men [62,63]. Finally, lysoPC (P-16:0), a lysophosphatidylcholine consisting of one chain of plasmalogen 16:0 at the C-1 position, was in lower amount in the seminal plasma of HF bulls. In humans, glycerophospholipids, including lysophosphatidylcholines, were found in higher concentrations in the seminal plasma of infertile men [64]. Interestingly, another lysophosphatidylcholine (i.e., lysoPC (0:0/18:2(9Z,12Z))) was higher in CRY-P compared to F-P.

Therefore, differences in the metabolites in fresh seminal plasma between HF and LF bulls allowed for the identification of potential fertility markers. However, in earlier studies, other fertility markers were identified in bovine fresh seminal plasma, such as 2-oxoglutaric acid and fructose [21], or citrate, taurine, isoleucine and leucine [15]. The differences among these studies are likely due to the different methods of analysis, as in earlier reports, gas chromatography–mass spectrometry [21] or 1H nuclear magnetic resonance were employed [15].

In this study, changes in metabolome between HF and LF bulls were also observed in diluted seminal plasma from cryopreserved semen (CRY-P). Some of the metabolites followed the same pattern described for fresh seminal plasma samples. Interestingly, the CRY-P of HF bulls also contained higher levels of GPC and lower levels of butyrylcarnitine, as in fresh seminal plasma. In addition, glycine betaine and l-carnitine contents were lower in the CRY-P of HF bulls. Metabolic relationships exist among the differentially expressed metabolites between HF and LF bulls, as GPC, glycine betaine and l-carnitine are involved in the same metabolic pathway. In addition, these metabolites have been proven to influence male fertility, as previously mentioned [41,52,62,63].

### 4.4. Metabolomic Analysis of Spermatozoa from HF and LF Bulls

The differences in metabolite content in fresh spermatozoa between HF and LF bulls were limited to two metabolites. Specifically, sperm from HF bulls contained higher levels of GPC and lysoPC (16:0). Glycerophosphocholines derive from unsaturated fatty acids that are the main components of the sperm membrane, essential for sperm viability and motility. It is known that sperm, during epididymal transit, incorporate GPC that is involved in sperm capacitation and metabolism [65]. Indeed, GPC may provide sperm energy substrates, such as l-glycerol 3 phosphate, also known to regulate sperm metabolism on capacitation [66]. The other identified metabolite accumulated in the sperm from HF bulls was lysoPC (16:0), a lysophospholipid with a chain of palmitic acid at the C-1 position. Phospholipids, lysophospholipids and fatty acids are implicated in several functions and play a role in sperm acrosome reaction, metabolism and motility [67]. Lysophosphatidylcholine, formed by the hydrolysis of phosphatidylcholine, a normal component of sperm plasma membrane, influences motility, capacitation and acrosome reaction [27,28], contributing to membrane-fusion events during fertilization [68]. In a previous study, other metabolites, such as gamma–aminobutyric acid, carbamate, benzoic acid, lactic acid and palmitic acid, were identified as potential bull fertility markers, but a different method of analysis (i.e., GS–MS) was employed [20].

Finally, differences in cryopreserved spermatozoa between HF and LF bulls were limited to glycine betaine, whose content was lower in the former bulls. This finding is difficult to interpret, and future in vitro studies are needed to better understand the reasons. The significantly higher proportion of live spermatozoa undoubtedly accounts for a more physiological sperm metabolism in HF bulls. In addition, spermatozoa from HF bulls exhibited improved membrane integrity, assessed by the HOS test that measures the cell ability to counteract osmotic stress. This may suggest a lower capacity of spermatozoa from LF bulls to respond to the stress conditions related to cryopreservation, to which they react with a compensatory increase in glycine betaine. On the other hand, we cannot rule out that the higher amount of glycine betaine recorded in LF bulls may be due to reduced utilization, in line with the lower proportion of viable spermatozoa. It is, however, interesting to note that higher levels of glycine betaine were also recorded in cryopreserved vs. fresh spermatozoa, with the former known to exhibit lower viability [30].

## 5. Conclusions

In conclusion, cryopreservation affects bovine semen metabolome at both plasmatic and cellular compartments, resulting in the variation of metabolites that counteract oxidative stress and regulate sperm capacitation and motility. Furthermore, the metabolic profiles differ between HF and LF bulls, particularly in fresh seminal plasma, with several lipids differentially expressed, indicating the way for the identification of potential fertility biomarkers. Differences in few metabolites between HF and LF bulls are also present in cryopreserved seminal plasma and spermatozoa. Due to the high individual variability in fertilizing ability among bulls, it is thought necessary to increase the sample size in order to validate these findings in future studies.

## Figures and Tables

**Table 1:** Values of *p* Corrected and Log2Fold Change of putatively identified metabolites in fresh and cryopreserved seminal plasma (F-P and CRY-P) and fresh and cryopreserved spermatozoa (F-S and, CRY-S) samples (*t*-test with *p*-value < 0.05 and fold change > 2.0).

		F-P vs. CRY-P		F-S vs. CRY-S	
Compound	Mass (Da)	Log2FC	*p*Corr	Log2FC	*p*Corr
**Glycine Betaine ^a^**	117.0789			1.68	3.43 × 10^−2^
**LysoPC** (**0:0/18:2**(**9Z,12Z**)) *^,a^	519.334	1.54	1.34 × 10^−31^		
**Pyro-l-glutaminyl-l-glutamine ^a^**	257.1015			1.26	3.49 × 10^−2^

* LysoPC (0:0/18:2(9Z,12Z)) lysophosphatidylcholine (0:0/18:2(9Z,12Z)). ^a^ Down-regulation against CRY group; blank cells mean the metabolite was not statistically different.

**Table 2:** Post-thawing morphological and functional sperm parameters in high fertility (HF, *n* = 4), and low fertility (LF, *n* = 4) bulls.

Parameters	HF (Mean ± SE)	LF (Mean ± SE)
Viability (%) *	80.0 ± 2.0 ^A^	46.7 ± 6.7 ^B^
Total Anomalies (%) *	7.8 ± 1.3	9.7 ± 2.5
HOS+ (%) *	67.8 ± 1.8 ^A^	55.0 ± 2.4 ^B^
Tunel + (%) *	10.5 ± 2.2	14.6 ± 1.9
Cleavage (%) **	75.3 ± 1.8 ^A^	54.9 ± 3.5 ^B^
Blastocysts (%) **	31.1 ± 1.8 ^A^	13.4 ± 2.8 ^B^

^A,B^ Values within columns with different superscripts are significantly different; *p* < 0.01. * Parameters were assessed on six ejaculates/bull (*n* = 48 ejaculates). ** In vitro fertilization was carried out with three randomly selected ejaculates/bull (*n* = 24).

**Table 3:** Values of *p* Corrected and Log2Fold Change of putatively identified metabolites in fresh and cryopreserved seminal plasma (F-P, CRY-P) and spermatozoa (F-S, CRY-S) samples in low and high fertility bulls (*t*-test with *p*-value < 0.05 and fold change > 2.0).

Compound	Mass (Da)	F-P		CRY-P		F-S		**CRY-S**	
		Log2FC	*p*Corr	Log2FC	*p*Corr	Log2FC	*p*Corr	Log2FC	*p*Corr
2,3-Diacetoxypropyl stearate ^B^	44.3283	1.43	2.13 × 10^−3^						
Glycine Betaine ^A^	117.0789			1.77	7.97 × 10^−32^			1.32	1.39 × 10^−2^
Butyrylcarnitine ^A^	231.1473	1.14	7.76 × 10^−3^	1.32	3.84 × 10^−3^				
Glycerol tripropanoate ^B^	260.1239	1.63	8.55 × 10^−3^						
GPC *^,^^B^	257.1032	1.07	1.31 × 10^−1^	1.54	8.73 × 10^−3^	2.23	3.95 × 10^−3^		
l-Acetylcarnitine ^B^	203.1159	1.00	6.04 × 10^−1^						
l-Carnitine ^A^	161.1049			1.38	4.60 × 10^−31^				
LysoPC (16:0/0:0) *^,B^	495.3346					2.38	1.20 × 10^−3^		
LysoPC (P-16:0/0:0) *^,A^	479.3382	2.04	1.61 × 10^−2^						
Piperidine ^A^	85.892	1.32	1.098 × 10^−5^						

* GPC: glycerophosphocholine, LysoPC (16:0/0:0): lysophosphatidylcholine (16:0/0:0), LysoPC (P-16:0/0:0): lysophosphatidylcholine (P-16:0/0:0). ^A^ Up-regulation in low- vs. high-fertility group; ^B^ Down-regulation in low- vs. high-fertility group; blank cells mean the metabolite was not statistically different.

## Supplementary Materials

The following are available online at https://www.mdpi.com/2076-2615/10/6/1065/s1, **Figure S1.** Hierarchical clustering analysis of fresh (in blue; F-P) and cryopreserved (in red; CRY-P) seminal plasma. Differentially expressed metabolites were obtained from *t*-test *p*-value < 0.05 and fold change > 2.0. **Figure S2.** Hierarchical clustering analysis of fresh (blue; F-S) and cryopreserved (red; CRY-S) spermatozoa. Differentially expressed metabolites were obtained from *t*-test *p*-value < 0.05 and fold change > 2.0. **Figure S3.** Hierarchical clustering analysis of fresh seminal plasma for high (in red; HF) and low (in blue; LF) fertility bulls. Differentially expressed metabolites were obtained from *t*-test *p*-value < 0.05 and fold change > 2.0. **Figure S4.** Hierarchical clustering analysis of cryopreserved seminal plasma for high (in red; HF) and low (in blue; LF) fertility bulls. Differentially expressed metabolites were obtained from *t*-test *p*-value < 0.05 and fold change > 2.0. **Figure S5.** Hierarchical clustering analysis of fresh spermatozoa for high (in red; HF) and low (in blue; LF) fertility bulls. Differentially expressed metabolites were obtained from *t*-test *p*-value < 0.05 and fold change > 2.0. **Figure S6.** Hierarchical clustering analysis of cryopreserved spermatozoa for high (in red; HF) and low (in blue; LF) fertility bulls. Differentially expressed metabolites were obtained from *t*-test *p*-value < 0.05 and fold change > 2.0.
