# Supplementary material for: Changes in Bull Semen Metabolome in Relation to Cryopreservation and Fertility
Source: Animals (Basel). 2020 Jun 19;10(6):1065. doi: 10.3390/ani10061065 (PMC7341314; doi:10.3390/ani10061065)
Supplement: Supplementary file 1 [file animals-10-01065-s001.pdf]

animals

Supplementary: Changes in Bull Semen Metabolome in Relation to Cryopreservation and Fertility

Valentina Longobardi [1], Michal A. Kosior [2], Nunzia Pagano [2], Gerardo Fatone [2,*], Alessia Staropoli [3], Anastasia Vassetti [3], Francesco Vinale [2,3], Giuseppe Campanile [2] and Bianca Gasparrini[2]

[1] Department of Precision Medicine, University of Campania Luigi Vanvitelli, Naples, 80138, Italy; ; longobardivalentina@gmail.com (V.L.);
[2] Department of Veterinary Medicine and Animal Production, Federico II University of Naples, 80137 Naples, Italym.kosior@hotmail.com (M.A.K.); nunzia91n@libero.it (N.P.); francesco.vinale@ipsp.cnr.it (F.V.); giucampa@unina.it (G.C.); bgasparr@unina.it (B.G.)
[3] CNR Institute for Sustainable Plant Protection, 80055 Portici, Italy; al.staropoli@gmail.com (A.S.); an.vassetti@libero.it (A.V.)
* Correspondence: gerardo.fatone@unina.it

Received: 1 June 2020; Accepted: 17 June 2020; Published: date

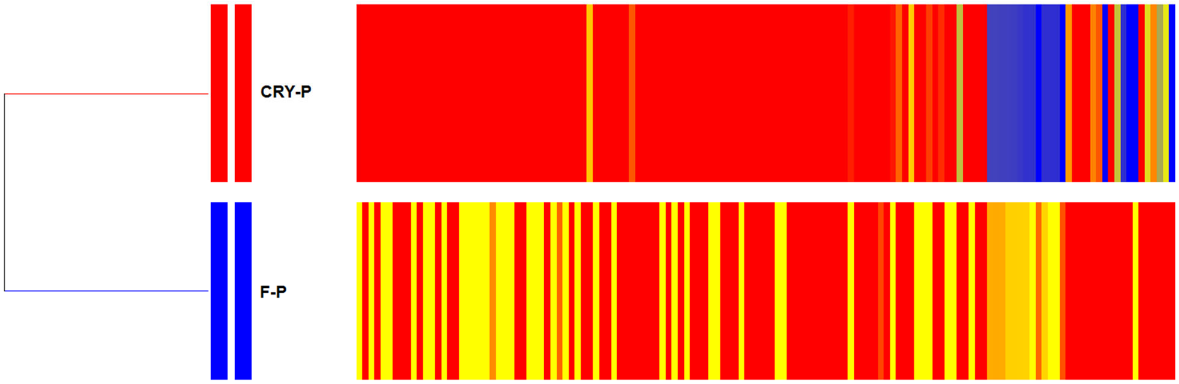

Figure S1. Hierarchical clustering analysis of fresh (in blue; F-P) and cryopreserved (in red; CRY-P) seminal plasma. Differentially expressed metabolites were obtained from T-test *p-value<0.05* and fold change>2.0.

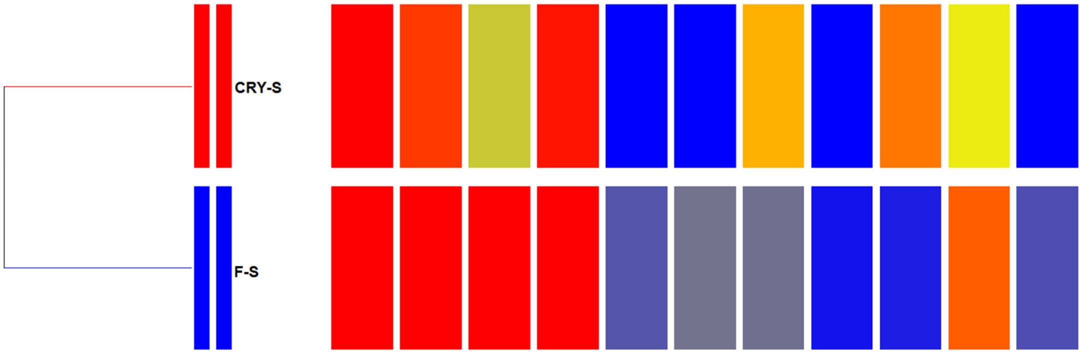

Figure S2. Hierarchical clustering analysis of fresh (blue; F-S) and cryopreserved (red; CRY-S) spermatozoa. Differentially expressed metabolites were obtained from T-test *p-value<0.05* and fold change>2.0.

animals

MDPI

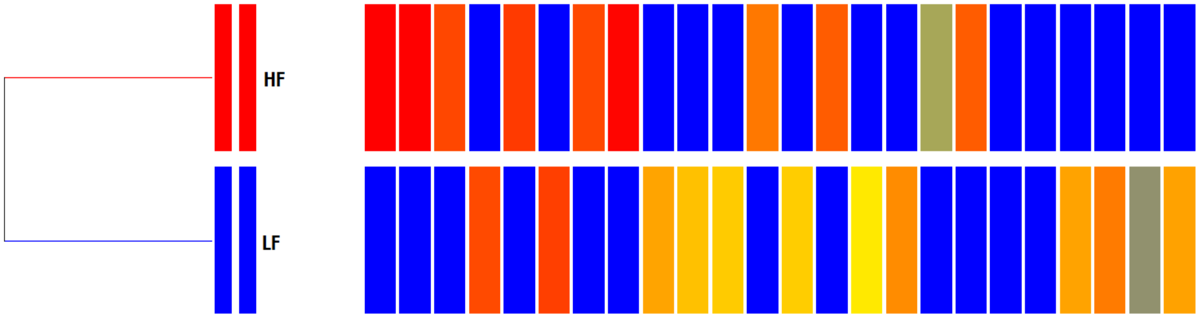

Figure S3. Hierarchical clustering analysis of fresh seminal plasma for high (in red; HF) and low (in blue; LF) fertility bulls. Differentially expressed metabolites were obtained from T-test *p-value<0.05* and fold change>2.0.

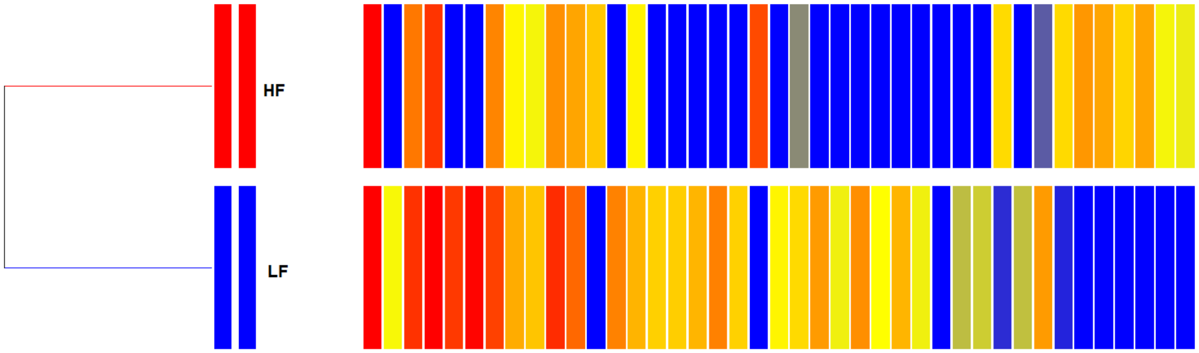

Figure S4. Hierarchical clustering analysis of cryopreserved seminal plasma for high (in red; HF) and low (in blue; LF) fertility bulls. Differentially expressed metabolites were obtained from T-test *p-value<0.05* and fold change>2.0.

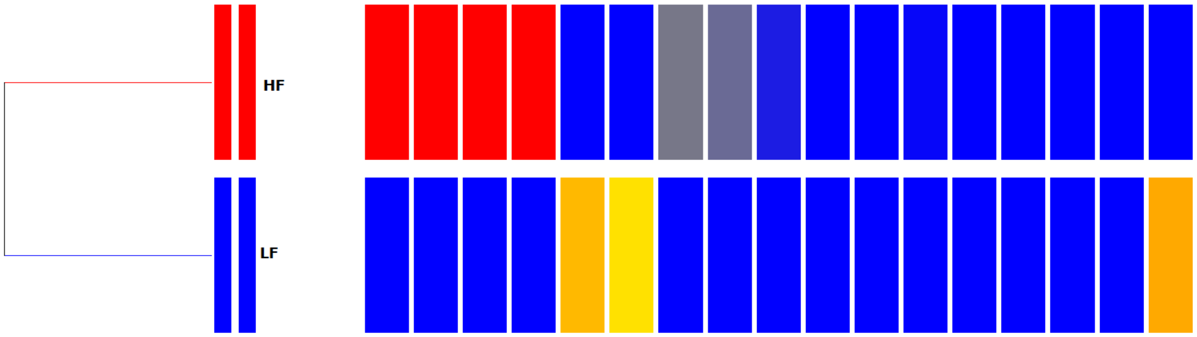

Figure S5. Hierarchical clustering analysis of fresh spermatozoa for high (in red; HF) and low (in blue; LF) fertility bulls. Differentially expressed metabolites were obtained from T-test *p-value<0.05* and fold change>2.0.

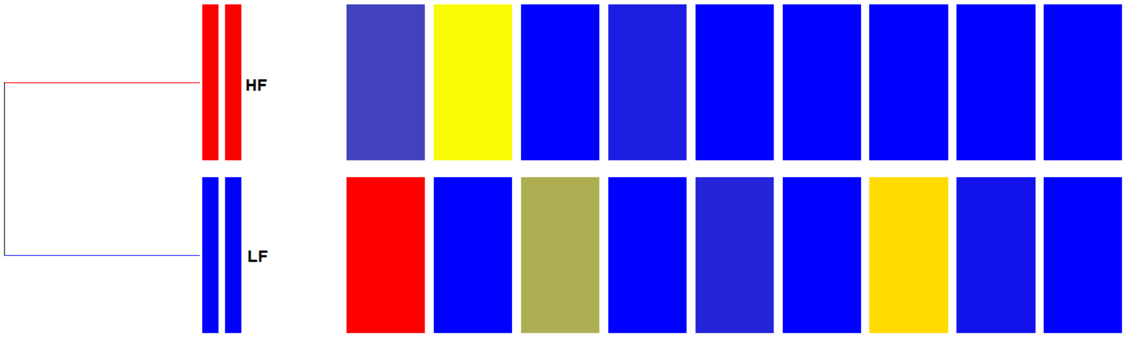

Figure S6. Hierarchical clustering analysis of cryopreserved spermatozoa for high (in red; HF) and low (in blue; LF) fertility bulls. Differentially expressed metabolites were obtained from T-test *p-value<0.05* and fold change>2.0.
